# Impaired muscle function, including its decline, is related to greater long‐term late‐life dementia risk in older women

**DOI:** 10.1002/jcsm.13227

**Published:** 2023-04-19

**Authors:** Simone Radavelli‐Bagatini, Helen Macpherson, David Scott, Robin M. Daly, Jonathan M. Hodgson, Simon M. Laws, Kun Zhu, Richard L. Prince, Joshua R. Lewis, Marc Sim

**Affiliations:** ^1^ Nutrition and Health Innovation Research Institute, School of Medical and Health Sciences Edith Cowan University Joondalup WA Australia; ^2^ Institute of Physical Activity and Nutrition Deakin University Geelong VIC Australia; ^3^ School of Clinical Sciences at Monash Health Monash University Clayton VIC Australia; ^4^ Medical School University of Western Australia Crawley WA Australia; ^5^ Centre for Precision Health Edith Cowan University Joondalup WA Australia; ^6^ Collaborative Genomics and Translation Group, School of Medical and Health Sciences Edith Cowan University Joondalup WA Australia; ^7^ Department of Endocrinology and Diabetes Sir Charles Gairdner Hospital Nedlands WA Australia; ^8^ School of Pharmacy and Biomedical Sciences, Faculty of Health Sciences, Curtin Health Innovation Research Institute Curtin University Bentley WA Australia; ^9^ Centre for Kidney Research, Children's Hospital at Westmead, School of Public Health, Sydney Medical School the University of Sydney Sydney NSW Australia

**Keywords:** Grip strength, Timed‐up‐and‐go, Cognitive function, Alzheimer's disease

## Abstract

**Background:**

Impaired muscle function has been identified as a risk factor for declining cognitive function and cardiovascular health, both of which are risk factors for late‐life dementia (after 80 years of age). We examined whether hand grip strength and timed‐up‐and‐go (TUG) performance, including their change over 5 years, were associated with late‐life dementia events in older women and whether any associations provided independent information to Apolipoprotein E _ℇ_4 (*APOE*_ℇ_4) genotype.

**Methods:**

Grip strength and TUG were assessed in community‐dwelling older women (mean ± SD; age 75.0 ± 2.6 years) at baseline (*n* = 1225) and 5 years (*n* = 1052). Incident 14.5‐year late‐life dementia events (dementia‐related hospitalization/death) were obtained from linked health records. Cardiovascular risk factors (Framingham Risk Score), *APOE* genotyping, prevalent atherosclerotic vascular disease and cardiovascular‐related medications were evaluated at baseline. These were included in multivariable‐adjusted Cox‐proportional hazards models assessing the relationship between muscle function measures and late‐life‐dementia events.

**Results:**

Over follow‐up, 207 (16.9%) women had a late‐life dementia event. Compared with women with the highest grip strength (Quartile [Q] 4, 25.8 kg), those with the lowest grip strength (Q1, 16.0 kg) had greater hazard for a late‐life dementia event (HR 2.27 95% CI 1.54–3.35, *P* < 0.001). For TUG, the slowest women (Q4, 12.4 vs. Q1, 7.4 s) also recorded a greater hazard for a late‐life dementia event (HR 2.10 95% CI 1.42–3.10, *P* = 002). Weak hand grip (<22 kg) or slow TUG (>10.2 s) provided independent information to the presence of an *APOE*_ℇ_4 allele (*n* = 280, 22.9%). Compared with women with no weakness and no *APOE*_ℇ_4 allele, those with weakness and *APOE*_ℇ_4 allele had a greater hazard (HR 3.19 95% CI 2.09–4.88, *P* < 0.001) for a late‐life dementia event. Women presenting with slowness and the *APOE*_ℇ_4 allele also recorded a greater hazard for a late‐life dementia event (HR 2.59 95% CI 1.64–4.09, *P* < 0.001). For 5‐year muscle function changes, compared with women with the lowest performance decrement (Q1), those with the largest decrement (Q4) had higher hazards for a late‐life dementia event (grip strength HR 1.94 95% CI 1.22–3.08, *P* = 0.006; TUG HR 2.52 95% CI 1.59–3.98, *P* < 0.001) over the next 9.5 years.

**Conclusions:**

Weaker grip strength and slower TUG, and a greater decline over 5 years, were significant risk factors for a late‐life‐dementia event in community‐dwelling older women, independent of lifestyle and genetic risk factors. Incorporating muscle function measures as part of dementia screening appears useful to identify high‐risk individuals who might benefit from primary prevention programmes.

## Introduction

Dementia is a global public health concern with increasing social and economic impacts. In 2018, dementia was estimated to cost $US1 trillion to the global economy, and this is projected to reach $US2 trillion by 2030. An estimated 50 million people live with dementia globally with this number projected to triple by 2050.^1^ An extended asymptomatic prodromal period of up to 20 years, characterized by gradual accumulation of neuropathological disease processes, is a common manifestation of dementia, in particular Alzheimer's disease (AD) including its related dementias.^2^ Late‐life dementias are those that occur after the age of 80 years, often characterized by a set of pathological processes that affect the size of the cortex and hippocampus (e.g. tauopathy, inflammation, synucleinopathy, amyloid aggregation and strokes). This form of dementia can be influenced by positive or negative consequences of environmental exposures (e.g. physical activity or obesity).^3^ As such, it is essential that populations at high risk for developing late‐life dementia are identified during this asymptomatic prodromal period for early inclusion into primary prevention strategies.

Drug treatments for dementia typically serve to control symptoms, as oppose to alter its progression,^4^ especially at the early stage.^5^ Clearly, there is a need to focus on environmental/lifestyle/behavioural factors that can slow the progression and/or prevent dementia. Indeed, the 2020 Lancet Commission on dementia prevention, intervention and care highlighted physical inactivity as a major modifiable risk factor for dementia.^6^ Recent work also suggests that compromised muscle function, including reduced muscle strength and poorer physical function, are linked with compromised cognitive function and its decline.^7^, ^8^ A scoping review (15 studies) including individuals over 60 years reported that weaker hand grip strength was associated with decline in cognition and incident dementia over time (ranging from 1 to 7 years).^7^ It has also been suggested that a decline in cognitive function may precede loss of strength.^7^ However, findings from 190 406 middle‐aged to older adults from the UK Biobank cohort, utilizing neuroimaging measures, indicated that observed associations between hand grip strength and cognitive, neuroimaging and dementia outcomes were not due to reverse causation.^9^ Because the UK Biobank sample was still relatively young, with a mean age of 56.5 years, it is less informative about dementia outcomes that occur later in life. Accelerated decline of gait speed is another feature of neurodegenerative diseases including dementia.^8^ Mobility, as measured by the timed‐up‐and‐go (TUG) test, has been associated with cognitive performance,^10^ although it may have limited predictive value when cognitive function remains stable over time.^11^ In terms of dementia incidence, impaired TUG performance at age 66 years has been associated with up to 34% and 65% greater relative hazards for total dementia and vascular dementia over 3.8 years, respectively.^12^

Collectively, the aforementioned findings may be pertinent to late‐life dementia due to its strong links with vascular and non‐vascular risk factors.^3^ Perhaps, the proposed link between impaired muscle strength with incidence of dementia may in part be mediated by vascular‐related mechanisms (and environment/lifestyle), rather than primarily genetic variants (e.g. Apolipoprotein E _ℇ_4; *APOE*_ℇ_4) that are commonly associated with AD,^9^ a greater cause of death for women than men after 80 years.^13^ Specifically, *APOE*_ℇ_4 has been implicated in the deposition of amyloid β, hyperphosphorylated tau tangles (neurofibrillary tangles) in the brain, lipid metabolism and vascular integrity/function that can severely disrupt brain blood flow.^14^ Previous work reporting that impaired muscle function is related to dementia has typically adopted younger cohorts (e.g. 56–66 years),^9^, ^12^ not considered genetic risk (e.g. *APOE*_ℇ_4),^15^ nor the concurrent change in strength and physical function over time. To our knowledge, no study has examined the relationship between muscle function and late‐life dementia. As such, the aim of this study was to determine the relationship of hand grip strength and TUG performance (and their changes over 5‐years) with long‐term risk for a late‐life dementia event (comprising any dementia‐related hospitalization or death) in community‐dwelling older women (>70 years) and whether any associations provided independent information to *APOE*_ℇ_4 genotype.

## Methods

### Study population

The population included individuals from the Perth Longitudinal Study of Aging in Women (PLSAW). Ambulant community‐dwelling older Australian women (≥70 years) were recruited in 1998 to a 5‐year, double‐blind, randomized placebo‐controlled trial of daily calcium supplementation to prevent fracture, the Calcium Intake Fracture Outcome Study (CAIFOS).^16^ Participants were included based on an expected survival beyond 5 years. Exclusion criterion was the use of any medications (including hormone replacement therapy) that could interfere with bone metabolism.^16^ Women from this study had a comparable medication and disease burden to that of the general population and had a Mini‐Mental State Examination (MMSE) score ≥25 upon study entry. However, socio‐economic status was higher than the general population.^16^ All women received 1.2 g of elemental calcium (calcium carbonate) or a matching placebo each day for 5 years. After the completion of CAIFOS, participants were subsequently enrolled in a further 10 years of observational follow‐up: the PLSAW (http://www.lsaw.com.au).

**Figure 1:**
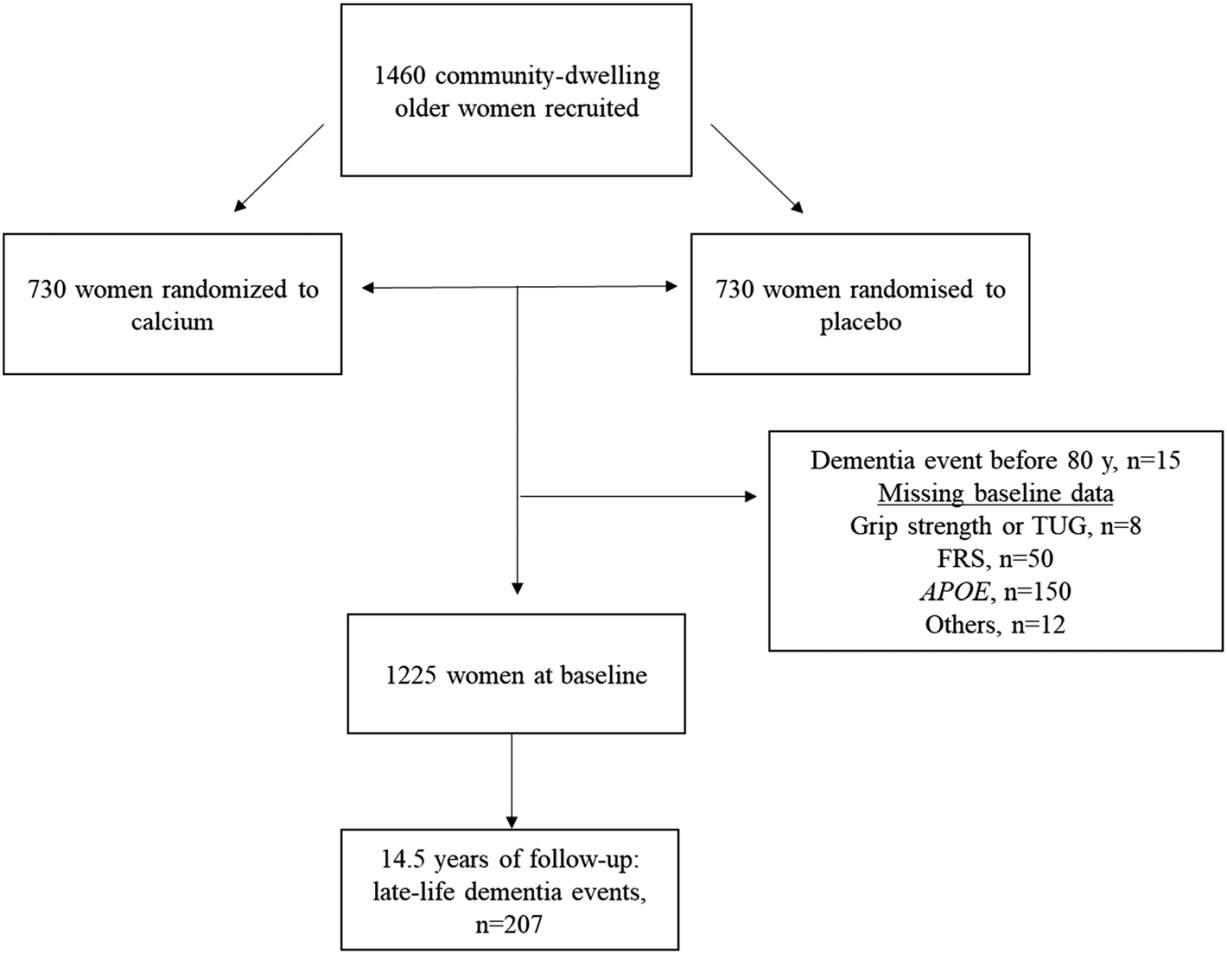
Participant flow chart. APOE, apolipoprotein E; FRS, General Framingham Risk Sore. Grip strength and timed‐up‐and‐go (TUG) was assessed in 1052 women at 5 years.

From the 1460 women recruited, cardiovascular risk factors used to compute the General Framingham Risk Score (FRS) and *APOE* genotypes were assessed in 1260 women. After excluding women with missing data (*n* = 20) and those who experienced a dementia event (including hospitalization or death) before the age of 80 years (*n* = 15), 1225 women were included in the study (*Figure* *1*). For all study participants, written informed consent was obtained, including follow‐up from electronic linked health records. Ethics approval was granted by the Human Ethics Committee of the University of Western Australia.

### Baseline risk factor assessment

Previous medical history and current use of medications were verified by each participants primary healthcare provider, where possible. The International Classification of Primary Care–Plus (ICPC‐Plus) method^[S1]^ was adopted, enabling aggregation of different terms for similar pathologic entities as defined by the ICD‐9 coding system. Information was used to determine prevalent diabetes (T89001‐90009) and atherosclerotic vascular disease (ASVD). Prevalent ASVD included coronary heart disease (ICD‐9‐CM codes 410‐414); heart failure (ICD‐9‐CM code 428); cerebrovascular disease excluding haemorrhage (ICD‐9‐CM codes 433‐438); and peripheral arterial disease (ICD‐9‐CM codes 440‐444).^[S2]^ Cardiovascular medications included antihypertensives, statins and low‐dose aspirin. Physical activity and smoking history were captured using questionnaires. Being a former/current smoker was defined as smoking >1 cigarette/day for >3 months at any time during the participants' life. Participants were asked about participation in sport, recreation and/or regular physical activities undertaken in the 3 months prior to their baseline visit, as described previously.^[S3]^ Briefly, the level of activity, expressed in expended kilojoules per day, was calculated from the questionnaire using a validated method applying the type of activity, time engaged in the activity and the participant's body weight. Weight (in kg) was assessed using digital scales. Height (in cm) was assessed using a stadiometer, and the body mass index (BMI) was calculated as kg/m^2^. Blood pressure was measured (average of three measurements) on the right arm with a mercury column manometer with participants seated upright and rested for 5 min. Genotyping for *APOE* was performed by polymerase chain reaction amplification with oligonucleotide primers.^[S4, S5]^ Alcohol intake was obtained from a questionnaire (none, <10 standard drinks per week or ≥10 standard drinks per week). The 10‐year estimated General Framingham Cardiovascular Risk Score (FRS) was calculated using BMI and included age, sex, previous diabetes, smoking status and systolic blood pressure.^[S6]^

### Muscle strength and physical function assessment

Measures of muscle function including hand grip strength and TUG were assessed at baseline (1998) and 5 years later (2003), as described previously.^17^ Briefly, a hand‐held dynamometer (Jamar Hand Dynamometer, Lafayette Instrument Company, USA) was used to assess grip strength (in kg). For TUG, this was the time taken (to the nearest tenth of a second) for an individual to rise from a chair, walk 3 m, turn around and to return to sit on the chair. Interobserver coefficient of variation error was 7% and 6%, respectively for grip strength and TUG in our laboratory, as assessed on a random sample of 30 women.

### Late‐life dementia outcomes

Dementia outcomes over 14.5 years were tracked through the Western Australian Data Linkage System (Department of Health Western Australia) and retrieved from the Western Australia Hospital Morbidity Data Collection (HMDC). Records were obtained for each participant from 1998 until 2013. Codes were identified using the International Classification of Diseases, Injuries and Causes of Death Clinical Modification (ICD‐9‐CM) or the International Statistical Classification of Diseases and Related Health Problems, 10^th^ Revision, Australian Modification (ICD‐10‐AM).^18^, ^19^ The primary outcome of the study was late‐life (after the age of 80 years) dementia events (hospitalization and/or death). This was undertaken as the inclusion of death data has been reported to improve identification of those with dementia.^[S7]^ Dementia codes included AD (ICD‐9‐CM 331.0, ICD‐10‐AM F00, G30), vascular dementias (ICD‐9‐CM 290.4, ICD‐10‐AM F01) and unspecified dementias (F03).^[S8]^ These were also considered individually as secondary outcomes. The principal or additional discharge diagnosis codes from hospital morbidity data collection was used to define events. To increase identification of dementia cases we used the linked coded multiple causes of death data (dementia codes as above) or parts 1 and 2 of the death certificate where coded cause of death data was not yet available. This methodology has been used to investigate the aetiology and consequences of dementia.^[S9]^

### Statistical analysis

The statistical analysis was performed using IBM SPSS Statistics for Windows, version 25.0 (IBM Corp., Armonk, NY, USA), Stata software, version 14 (StataCorp LLC, College Station, Texas, USA) and R software (version 3.4.2, R Foundation for Statistical Computing, Vienna, Austria).^[S10]^ Baseline characteristic comparisons for women who did not present versus presented with a late‐life dementia event were obtained using one‐way ANOVA, Mann–Whitney *U* test or Pearson's chi‐square where appropriate. Cox proportional hazards modelling were used to investigate the relationship between grip strength and TUG (as separate exposures) and late‐life dementia outcomes including (i) events (hospitalization and/or death), (ii) hospitalizations and (iii) deaths. Global tests (estat phtest) indicated proportional hazards assumptions were not violated when considering the relationships between grip strength or TUG and all late‐life dementia outcomes. These relationships are presented graphically using the ‘effects’ R package.^[S11]^ Hazard ratios (HRs) and 95% confidence intervals (CIs) were obtained from the model with grip strength or TUG fitted as a continuous variable through a restricted cubic spline using the ‘rms’ R package.^[S12]^ HR estimates were graphed and calculated relative to a reference value being the median of Quartile 4 (Q) for grip strength and Q1 for TUG whilst being plotted against the outcome variable, with 95% CIs provided. *P*‐values for HRs were obtained using Wald tests. The x‐axis was truncated at 3 SD above the mean for visual simplicity only for all graphs. Cox proportional hazards modelling was also used to examine the influence of binary exposures (weak grip strength [<22 kg] and slow TUG [>10.2 s]) with all late‐life dementia outcomes. Cut‐points for weak grip strength (<22 kg) and slow TUG were (>10.2 s) were selected due to their potential association with adverse health outcomes (e.g. weakness, risk of falling, fractures).^[S13, S14]^ For all regression analysis, we considered two models (i) unadjusted and (ii) multivariable adjusted: treatment code (calcium or placebo), FRS, cardiovascular medications (statins and low‐dose aspirin), prevalent ASVD, *APOE* genotypes (_ℇ_2/3, _ℇ_2/4, _ℇ_3/3, _ℇ_3/4, _ℇ_4/4), physical activity and alcohol intake.

### Additional analysis

We undertook analysis replacing FRS [estimate of cardiovascular disease (CVD) risk] with the individual variables used to derive the computed score (age, BMI, previous diabetes, smoking history and systolic blood pressure) to the multivariable‐adjusted model. Considering the advanced age of our cohort, competing risks analyses based on Fine and Gray's proportional subhazards model^[S15]^ to account for the competing risk of non‐dementia mortality was also undertaken. We also undertook analysis where both grip strength and TUG were simultaneously included (as continuous variables) as part of multivariable‐adjusted analysis with late‐life dementia events. Carrying the *APOE*_ℇ_4 allele is known to be associated with increased risk of dementia.^3^ As such, the relationship between grip strength, TUG and late‐life dementia events in carriers and non‐carriers of the *APOE*_ℇ_4 allele was investigated separately. We also dichotomized grip strength or TUG and *APOE*_ℇ_4 allele into four groups. For grip strength, these included (i) no weakness [grip strength >22 kg] and no *APOE*_ℇ_4 allele; (ii) no weakness and an *APOE*_ℇ_4 allele; (iii) weakness [grip strength ≤22 kg] and no *APOE*_ℇ_4 allele; and (iv) weakness and *APOE*_ℇ_4 allele. For TUG, these four groups included (i) no slowness [TUG ≤10.2 s] and no *APOE*_ℇ_4 allele; (ii) no slowness and an *APOE*_ℇ_4 allele; (iii) slowness [TUG >10.2 s] and no *APOE*_ℇ_4 allele; and (iv) slowness and *APOE*_ℇ_4 allele. Finally, in women who also undertook muscle strength and physical function tests at year 5 (*n* = 1052), we explored the relationship between change in grip strength and TUG (continuously and by Q) over 5 years (1998–2003) with late‐life dementia outcomes over the next 9.5 years. Here, based on the 5‐year change in grip strength and TUG, these women then categorized into Q based on the decrement in performance for each test; Q1, smallest change; Q2, some change; Q3, moderate change; and Q4, largest change. These analyses were limited to women who did not present with a late‐life dementia event between 1998 and 2003.

## Results

Participant flowchart is presented in *Figure* *1*. Baseline characteristics of participants are displayed in *Table* *1*, whilst comparison with those who were excluded is presented in *Table* *S1*. At baseline, women who experienced a late‐life dementia event tended to be slightly older, have a lower BMI, were a smoker and had a higher FRS, weaker hand grip strength and slower TUG. A higher proportion of these women also had a history of smoking and presented with the *APOE*_ℇ_4 allele (*n* = 280, 22.9%).

**Table 1:** Baseline characteristics of the study population stratified by development of late‐life dementia

	Whole cohort	No late‐life dementia	Late‐life dementia	*P*‐value
Number (%)	1225	1018 (83.1)	207 (18.5)	
Age, years	75.1 ± 2.7	75.0 ± 2.6	76.1 ± 2.8	<0.001
Body mass index, kg/m^2^	27.2 ± 4.6	27.4 ± 4.6	26.4 ± 4.5	0.006
Ever smoked, yes (%)	440 (35.9)	352 (34.6)	88 (42.5)	0.029
Systolic blood pressure, mmHg	138 ± 18	138 ± 18	138 ± 21	0.699
Antihypertensive medication, yes (%)	523 (42.7)	436 (42.8)	87 (42.0)	0.832
Diabetes, yes (%)	73 (6.0)	53 (5.2)	20 (9.7)	0.014
Estimated CVD risk (Framingham), %	22.1 ± 11.0	21.8 ± 10.6	23.5 ± 12.6	0.044
Statins medication, yes (%)	234 (19.1)	196 (19.3)	38 (18.4)	0.765
Low‐dose aspirin, yes (%)	245 (22.0)	195 (19.2)	50 (24.2)	0.101
Previous ASVD, yes (%)	140 (11.4)	115 (11.3)	25 (12.1)	0.748
Physical activity, kcal	113 (36–204)	113 (36–202)	108 (42–208)	0.920
Alcohol, n (%)				0.416
*None*	233 (19.0)	187 (18.4)	46 (22.2)	
*<10 standard drinks p/w*	792 (64.7)	662 (65.0)	130 (62.8)	
*≥10 standard drinks p/w*	200 (16.3)	169 (16.6)	31 (15.0)	
Randomization, calcium (%)	615 (50.2)	516 (50.7)	99 (47.8)	0.453
*APOE* genotypes, yes (%)				<0.001
*APOE*_*ℇ*_ *2/3*	193 (15.8)	173 (17.0)	20 (9.7)	
*APOE*_*ℇ*_ *2/4*	27 (2.2)	23 (2.3)	4 (1.9)	
*APOE*_*ℇ*_ *3/3*	752 (61.4)	633 (62.2)	119 (57.5)	
*APOE*_*ℇ*_ *3/4*	235 (19.2)	179 (17.6)	56 (27.1)	
*APOE*_*ℇ*_ *4/4*	18 (1.5)	10 (1.0)	8 (3.9)	
Grip strength (kg)	20.6 ± 4.6	20.9 ± 4.5	19.1 ± 4.8	<0.001
Timed‐up‐and‐go (s)	9.3 (8.1–11.0)	9.2 (8.0–10.9)	9.8 (8.4–11.3)	0.002

Data expressed as mean ± SD, median (IQR) or number (%).

*APOE*, apolipoprotein E; ASVD, atherosclerotic vascular disease; mmHg, millimetres mercury.

### Muscle function and late‐life dementia over 14.5 years

**Figure 2:**
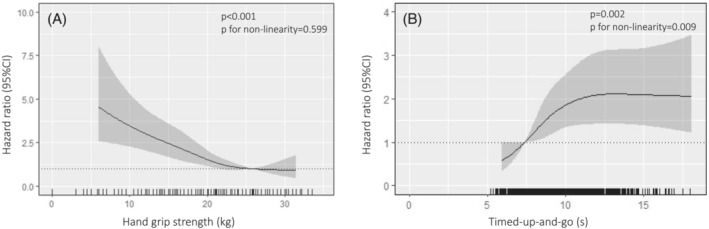
Restricted cubic splines based on multivariable‐adjusted Cox proportional hazards analysis highlighting the relative hazard between (A) hand grip strength and (B) timed‐up‐and‐go with any late‐life dementia event (hospitalization/death) over 14.5 years. Shaded represent 95% confidence intervals. Rug plot on the x‐axis represents an observation. The reference value represents the median hand grip strength (25.8 kg) and timed‐up‐and‐go (7.4 s) for the strongest (Quartile 4) and fastest women (Quartile 1), respectively.

For a late‐life dementia hospitalization (15 155 total person years) and/or death (15 568 total person years), the mean ± SD follow‐up was 12.4 ± 3.3 and 12.7 ± 3.1 years, respectively. A diagrammatic representation for the near‐linear relationship (p for nonlinearity = 0.599) between hand grip strength and late‐life dementia events (*P* < 0.001) is presented in *Figure* *2* *A*. In the multivariable‐adjusted analysis, compared with women with the highest grip strength (Q4, 25.8 kg), those with the lowest grip strength (Q1, 16.0 kg) had higher hazards for a late‐life dementia event (2.27 times), hospitalization (2.26 times) or death (2.45 times) (*Table* *2*). Similarly, women with weak grip strength (<22 kg, *n* = 744, 60.7%) presented with higher relative hazards for a late‐life dementia event (1.71 times), hospitalization (1.76 times) or death (1.62 times) (*Table* *S2*) compared with women with higher grip strength (≥22 kg), in the multivariable‐adjusted analysis.

**Table 2:** Hazard ratios (95% CI) for any late‐life dementia events, hospitalizations and deaths over 14.5 years by quartiles of hand grip strength

			Quartiles for hand grip strength^a^			
	Events		Quartile 1	Quartile 2	Quartile 3	Quartile 4
*Late‐life dementia events*	*207 (16.9)*	*Unadjusted*	**2.34 (1.59–3.42)**	**1.68 (1.23–2.30)**	**1.28 (1.00–1.64)**	1.00 (ref)
		*Adjusted*	**2.27 (1.54–3.35)**	**1.63 (1.19–2.24)**	1.24 (0.96–1.58)	1.00 (ref)
*Late‐life dementia hospitalizations*	*183 (14.9)*	*Unadjusted*	**2.30 (1.54–3.45)**	**1.63 (1.18–2.26)**	1.23 (0.95–1.59)	1.00 (ref)
		*Adjusted*	**2.26 (1.50–3.41)**	**1.59 (1.14–2.21)**	1.18 (0.92–1.52)	1.00 (ref)
*Late‐life dementia deaths*	*83 (6.8)*	*Unadjusted*	**2.60 (1.40–4.85)**	**1.84 (1.09–3.10)**	1.40 (0.90–2.17)	1.00 (ref)
		*Adjusted*	**2.45 (1.31–4.60)**	**1.73 (1.02–2.94)**	1.34 (0.86–2.09)	1.00 (ref)

^a^ Estimated hazard ratios and 95% CI from Cox proportional hazards analysis comparing the median hand grip strength from each quartile compared to Quartile 4. Median hand grip strength for Quartiles 1–4 was 16, 19.5, 22 and 25.8 kg, respectively. Bolded indicates *P* ≤ 0.05 compared to Quartile 4. Multivariable‐adjusted model includes general Framingham Risk Score, treatment code (calcium or placebo), alcohol intake, prevalent atherosclerotic vascular disease, prescription of statin medications, use of low‐dose aspirin, physical activity and apolipoprotein E genotype.

The multivariable‐adjusted non‐linear relationship (*P* for nonlinearity = 0.009) between TUG and late‐life dementia events (*P* = 0.002) is presented in *Figure* *2* *B*. Compared with women with the fastest TUG (Q1, 7.4 s), those with the slowest TUG (Q4, 12.4 s) had higher relative hazards for a late‐life dementia event (2.10 times), hospitalization (2.13 times) and/or death (2.98 times), respectively (*Table* *2*). Similarly, women with slow TUG (>10.2 s) had higher relative hazards for a late‐life dementia event (1.54 times), hospitalization (1.54 times) and/or death (1.91 times). When considering women with poor overall muscle function (weak grip strength plus slow TUG, *n* = 317, 25.9%), these individuals recorded higher relative hazards for a late‐life dementia event (1.72 times), hospitalization (1.71 times) and/or deaths (2.03 times) (*Table* S2). For the different types of late‐life dementias, 97 recorded AD, and 148 women recorded an unspecified late‐life dementia event. When considering AD events, compared with women with higher grip strength, those with weak grip strength recorded higher hazards (HR 1.79 95% CI 1.14–2.82, *P* = 0.011). This was not observed for slow TUG (HR 1.28 95% CI 0.84–1.95, *P* = 0.257). However, for unspecified dementia events, women with weak grip strength (HR 1.80 95% CI 1.25–2.60, *P* = 0.002) and slow TUG (HR 1.75 95% CI 1.26–2.45, *P* = 0.001) recorded higher relative hazards. Low numbers of women recorded vascular (*n* = 18) and other causes of late‐life dementia (*n* = 8); hence, no further analysis for these outcomes were performed.

### Additional analyses

For analysis where the individual measures (age, BMI, prevalent diabetes, SBP) used to compute the FRS were added to the multivariable‐adjusted model, the results (*Table* *S3*) were comparable to the primary analysis (*Table* *2*). Noteworthy, FRS was independently associated with late‐life dementia events (*P* < 0.05) in all of the primary analysis considering muscle function measures presented in *Tables* *2* *and* *3*. Specifically, for late‐life dementia events, compared with women with the highest grip strength (Q4), individuals with the lowest grip strength (Q1) had a 1.95 times higher relative hazard. For TUG, the slowest women (Q4) had 2.17 times higher relative hazard for a late‐life dementia event. Competing risks analyses for non‐dementia deaths did not substantially affect the point estimates for quartiles of grip strength and TUG (*Table* *S4*). When both grip strength and TUG were included simultaneously as part of multivariable‐adjusted analysis, grip strength (per kg decrease, HR 1.08 95% CI 1.05–1.11, *P* < 0.001) but not TUG was associated with lower risk for a late‐life dementia event. In women who carried an *APOE*_ℇ_4 allele (*n* = 280), those with the lowest grip strength (Q1) had 2.2 times higher hazard for a late‐life dementia event (*Table* *S5*), compared with women with the highest grip strength (Q4). For TUG, women with slower TUG (Q2, Q3, Q4) had 1.69–1.99 times greater hazards for a late‐life dementia event. Similarly, for women who did not carry the *APOE*_ℇ_4 allele (*n* = 945), those with the lower grip strength (Q1, Q2, Q3) compared with Q4 recorded between 1.39 and 2.47 times lower hazards for a late‐life dementia event. For TUG, slower women (Q3, Q4) recorded between 1.81 and 2.12 times higher relative hazard for a late‐life dementia event.

**Table 3:** Hazard ratios (95% CI) for any late‐life dementia events, hospitalizations and deaths over 14.5 years by quartiles of timed‐up‐and‐go time.

		Quartiles for timed‐up‐and‐go performance^a^			
		Quartile 1	Quartile 2	Quartile 3	Quartile 4
*Late‐life dementia events*	*Unadjusted*	1.00 (ref)	**1.48 (1.10–1.99)**	**1.87 (1.36–2.58)**	**2.17 (1.48–3.17)**
	*Adjusted*	1.00 (ref)	**1.50 (1.11–2.01)**	**1.86 (1.35–2.58)**	**2.10 (1.42–3.10)**
*Late‐life dementia hospitalizations*	*Unadjusted*	1.00 (ref)	**1.47 (1.07–2.01)**	**1.88 (1.34–2.65)**	**2.18 (1.45–3.27)**
	*Adjusted*	1.00 (ref)	**1.49 (1.09–2.04)**	**1.88 (1.33–2.65)**	**2.13 (1.41–3.22)**
*Late‐life dementia deaths*	*Unadjusted*	1.00 (ref)	**2.08 (1.15–3.77)**	**2.69 (1.44–5.04)**	**3.08 (1.57–6.05)**
	*Adjusted*	1.00 (ref)	**2.09 (1.15–3.79)**	**2.66 (1.42–5.01)**	**2.98 (1.50–5.90)**

^a^ Estimated hazard ratios and 95% CI from Cox proportional hazards analysis comparing the median timed‐up‐and‐go (TUG) time from each quartile compared to Quartile 1. Median TUG for Quartiles 1–4 was 7.4, 8.7, 10.1 and 12.4 s, respectively. Bolded indicates *P* ≤ 0.05 compared to Quartile 1. Multivariable‐adjusted model includes general Framingham Risk Score, treatment code (calcium or placebo), alcohol intake, prevalent atherosclerotic vascular disease, prescription of statin medications, use of low‐dose aspirin, physical activity and apolipoprotein E genotype.

**Figure 3:**
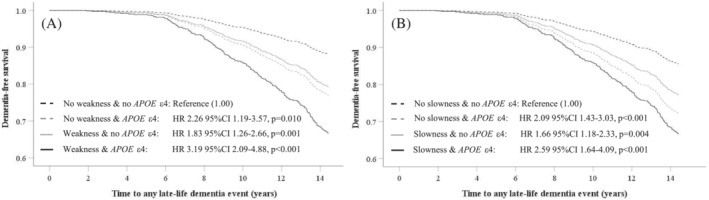
Multivariable‐adjusted Cox proportional hazards analysis for late‐life dementia events (hospitalization and/or death) dichotomized by weak hand grip strength (A) or slow timed‐up‐up‐and‐go (B) in‐conjunction with/without the presence of the apolipoprotein E ℇ4 (*APOE* ε4).

In analysis where the entire cohort was dichotomized based on grip strength and *APOE*_ℇ_4 allele into four groups (*Figure* *3*), compared with women with no *APOE*_ℇ_4 and higher grip strength (*n* = 379), women with weak grip strength and *APOE*_ℇ_4 (*n* = 179) had 3.19 times higher hazard for a late‐life dementia event. Women with higher grip strength with the *APOE*_ℇ_4 (*n* = 101) and those with weak grip strength but no *APOE*_ℇ_4 (*n* = 566) recorded 2.26 and 1.83 times higher relative hazards, respectively (*Figure* *3* *A*). Women with slow TUG and *APOE*_ℇ_4 (*n* = 97) recorded 2.59 times higher relative hazards for a late‐life dementia event compared with individuals with faster TUG and no *APOE*_ℇ_4 (*n* = 615) (*Figure* *3* *B*). Women with faster TUG with the *APOE*_ℇ_4 (*n* = 183) and those with slow TUG but no *APOE*_ℇ_4 (*n* = 330) recorded 2.09 and 1.66 times higher relative hazards, respectively.

### Change in muscle function over 5 years

A total of 1052 women were included in all analysis considering 5‐year change in muscle function. Mean change (95% CI) in grip strength and TUG over 5 years was −3.1 (−3.4, −2.9) kg and 1.6 (1.4, 1.8) s, respectively. Each kg decrease in grip strength or second increase in TUG was associated with between 7% and 9% greater relative hazard for a late‐life dementia event, hospitalization or death (*Table* *S6* *)*. Compared with women with the lowest decrement (Q1) in grip strength, those with the largest decrement (Q4) had higher hazards for a late‐life dementia event and hospitalization (1.92 and 2.32 times, respectively) (*Table* *4*). For late‐life dementia deaths, statistical significance was slightly attenuated for Q4 (*P* = 0.054) but not Q3 (*P* = 0.036). Baseline grip strength (per kg) remained significantly associated (all *P* < 0.001) with late‐life dementia events (HR 1.11 95% CI 1.07–1.15), hospitalizations (HR 1.12 95% CI 1.07–1.16) and deaths (HR 1.13 95% CI 1.06–1.20) as part of these analyses. For TUG, compared with women with the least change in TUG over 5 years (Q1), those with the largest increase in TUG (Q4) had 2.52, 2.67 and 4.11 times higher hazards for a late‐life dementia event, hospitalization and death, respectively (*Table* *4*). Baseline TUG (per sec) remained significantly associated (all *P* ≤ 0.001) with late‐life dementia events (HR 1.07 95% CI 1.03–1.10), hospitalizations (HR 1.07 95% CI 1.03–1.11) and deaths (HR 1.09 95% CI 1.03–1.14) as part of these analyses.

**Table 4:** Multivariable‐adjusted hazard ratios (95% CI) for late‐life dementia outcomes over 9.5 years presented by quartiles of change in grip strength or timed‐up‐and‐go (TUG) over 5 years (1998–2003) in 1052 women

			Quartiles of change in grip strength^a^ or timed‐up‐and‐go^b^			
	Events over 9.5 years	5‐year change	Quartile 1	Quartile 2	Quartile 3	Quartile 4
*Late‐life dementia events*	*168 (15.9)*	*Grip strength*	1.00 (ref)	1.28 (0.84–1.97)	1.19 (0.68–1.74)	**1.92 (1.21–3.05)**
		*TUG*	1.00 (ref)	1.16 (0.70–1.93)	1.60 (0.99–2.60)	**2.52 (1.59–3.98)**
*Late‐life dementia hospitalisations*	*149 (14.1)*	*Grip strength*	1.00 (ref)	1.48 (0.93–2.35)	1.18 (0.70–2.1.96)	**2.32 (1.41–3.82)**
		*TUG*	1.00 (ref)	1.33 (0.77–2.29)	**1.75 (1.03–2.95)**	**2.67 (1.62–4.40)**
*Late‐life dementia deaths*	*66 (6.3)*	*Grip strength*	1.00 (ref)	1.15 (0.54–2.45)	**2.16 (1.06–4.40)**	2.16 (0.99–4.72)
		*TUG*	1.00 (ref)	1.41 (0.58–3.43)	2.22 (0.97–5.10)	**4.11 (1.90–8.89)**

^a^ Range for Quartiles 1–4 for grip strength was < −0.8 kg, −0.8 to < −3.0 kg, −3.0 to < −5.5 kg and ≥ − 5.5 kg, respectively.

^b^ Range for Quartiles 1–4 for TUG was <0.02 s, 0.02 to <1.33 s, 1.33 s to <2.75 s, and ≥2.75 s, respectively. Multivariable‐adjusted model includes Framingham risk score, treatment code (calcium or placebo), alcohol intake, prevalent ASVD, prescription of statin medications, use of low‐dose aspirin, physical activity, apolipoprotein E genotype and baseline grip strength or TUG (where appropriate).

## Discussion

Our results indicate that lower hand grip strength and/or slower TUG (including their decline over 5 years) identified women with the greatest late‐life dementia risk, independent of and complementary to genetic (e.g. *APOE*_ℇ_4) as well as cardiovascular risk factors (e.g. FRS and ASVD). Consequently, physical assessment could be considered for use to assist clinicians screen individuals at higher risk of late‐life dementia for direction towards dementia‐related primary and secondary prevention strategies.^20^

Independent of lifestyle factors (e.g. smoking, physical activity, alcohol intake), we observed a 2.3–2.5 times higher hazard for a late‐life dementia event, hospitalization or death, in women with the lowest compared with the highest grip strength (Q1, 16.0 kg vs. Q4, 25.8 kg). These findings complement a study of 466 788 younger individuals (median age 56.5 years, 54.5% women) from the UK Biobank where those with the lowest hand grip strength had 72% and 87% higher risk of dementia incidence and mortality over ~9 years, respectively, compared with the strongest individuals.^15^ Unlike our study, genetic risk (e.g*. APOE*) was not considered. Another study including 2288 participants over the age of 65 years from the Adult Changes in Thought study also observed an association between higher hand grip strength (per kg) and lower risk of dementia (HR 0.87 95% CI 0.77; 0.99) almost 6 years later.^21^ When considering change in grip strength over time, a study in community‐dwelling Japanese men and women (*n* = 1055, mean age 68 years without dementia) reported those with the greatest decline in grip strength (≥15%) over 15 years had 51% higher risk for any dementia compared with those with unchanged grip strength.^22^ Collectively, such findings are comparable to our results where women with the largest 5‐year decline in grip strength (Q1) recorded between 1.9 and 2.4 times greater risk for a late‐life dementia event or hospitalization over the next 9.5 years. Here, it is important to highlight that our study is the first to focus on late‐life dementia in community‐dwelling older women (mean age ~75 years). Furthermore, the aforementioned findings are supported by a scoping review (15 studies with participants over the age of 60 years),^7^ which found that grip strength may be used as a surrogate marker to monitor changes in cognitive function over time, a major risk factor for dementia. Possibly due to a range of underlying shared pathologies, grip strength may present as a surrogate measure of CVD, inflammation and frailty,^23^ which are known risk factors for dementia.^6^ Grip strength could be a discriminating measure of neurological function and brain health due to overlapping neural basis of cognitive and motor decline.^24^ Data from over 40 000 people aged 40–70 years from the UK Biobank^25^ point to a potential role of brain regions vulnerable to neuropathology in AD,^26^ with volume of subcortical, hippocampal regions and temporal cortices associated with grip strength. In the context of CVD, a major contributor to vascular damage to white matter in the brain,^27^ the presence (and severity) of carotid plaques^28^ and calcification of the abdominal aorta^29^ has also been associated with poorer grip strength, the latter reported as a risk factor for late‐life dementia.^30^

We also observed that women with the slowest TUG (Q4, 12.4 s) had twofold to threefold higher relative hazard for a late‐life dementia‐related event, hospitalization and/or death, compared with those with the fastest TUG performance (Q1, 7.4 s). Most importantly, we report that women with the greatest decrement in TUG performance over 5 years recorded between 2.5 and 4.1 times greater risk for a late‐life dementia event or death. Likewise, in 3663 older individuals (mean age 73.5 years) who were free from dementia at baseline and followed over 9 years, slower gait speed was associated with 59% increased hazard of dementia.^31^ Of importance, the gait assessments obtained 4 and 7 years prior to dementia onset were also associated with an increased risk (46% and 30%, respectively). Noteworthy, in comparison to gait speed, TUG involves a more complex test incorporating manoeuvres typically used in daily life such as balance, walking and transferring (e.g. sitting and standing from a chair).^32^ More complex functional tasks may have greater potential to identify individuals at risk for dementia because cognitive and motor function share common neural substrates.^8^, ^33^ It is possible that the added complexity of the task translates to slow TUG time which may serve as a symptom of predementia (e.g. declining cognitive function). Unsurprisingly, a dual decline in both gait speed and cognition (specifically memory) has also been reported to have the strongest association with dementia risk in 16 855 individuals (mean age 75 years, 56% women).^34^ The aforementioned findings are supported by a scoping review including 39 studies (*n* = 57 456) advocating the evaluation of physical function via gait speed as beneficial in the clinical environment for dementia risk assessment.^8^ When considering a combination of muscle weakness and poor physical function, these measures are likely to improve risk stratification, as indicated by a higher relative hazard (between 1.6 and 2.0 times) for late‐life dementia outcomes.

Concurrent decline to cognitive and physical function is considered to be a result of dysregulation across multiple cellular processes such as genetic alterations, nutrient and lipid metabolism and elevated pro‐inflammatory proteins.^35^ For post‐menopausal women, lower circulating oestrogen levels may be exacerbated in vascular cognitive impairment and dementia pathogenesis.^36^ Whilst we identified the poorest physical function (including 5‐year decline) was associated with the greatest dementia risk in women, further research is needed to examine the role of events specific to women (pregnancy, menopause and sex hormones) and sex‐specific risk profiles for vascular disorders^37^ in the pathways leading to cognitive and physical decline. In regard to potential mechanisms, components of the TUG test (e.g. slow gait speed) may capture a range of unhealthy behaviours (e.g. poor diet and physical activity levels, smoking), as well as CVD,^38^ which increase the risk of dementia. Risk factors for compromised vasculature have also been suggested to increase vascular lesions of the brain (e.g. stroke and white matter lesions) that are capable of compromising motor control due to disrupted neuronal circuits.^31^ Noteworthy, higher grip strength has also been associated with increased white matter hyperintensity volume,^9^ which may be influenced by the vasculature, thus having strong implications for late‐life dementia. Unsurprisingly, endothelial dysfunction is a common mechanism implicated in vascular dementia and AD^39^ as well as poor muscle function and related phenotypes including sarcopenia and frailty.^40^ Collectively, a greater decline in grip strength and/or TUG, such as those observed over 5 years in our study, is likely to reflect a systematic exacerbation of the aforementioned risk factors that together increases dementia risk.

From a feasibility perspective, both grip strength and TUG are inexpensive and simple screening tools that can be used to identify individuals at a community level with impaired function. This information in conjunction with medical history can be considered by clinicians to determine if further assessment of dementia risk is required (e.g. genetics, cognitive function). Future work should seek to determine if the addition of such tests to current clinical dementia assessment tools substantially improves risk stratification. Under clinical guidance, early screening would also offer patients awareness around primary prevention strategies aiming to eliminate, reverse or attenuate dementia‐related risk factors. To this end, muscle function tests may be considered as an informative risk factor for a range of negative health outcomes including dementia.

This study has several strengths, including a large representative sample of community‐dwelling older women from the PLSAW cohort. For example, the prevalence of *APOE*_ℇ_4 allele in our cohort was 22.9%, which is comparable to the 22–28% reported in other Australian populations.^41^, ^42^ Furthermore, we demonstrate that grip strength and TUG provided independent information to genetic risk (e.g. *APOE*_ℇ_4) when considering late‐life dementia. Of importance, in the subgroup analysis where we stratify women by APOE ℇ4 and function, results demonstrate the importance of considering weakness and slowness irrespective of genetic risk. However, it is important to highlight the nature of this analysis is exploratory and hypothesis generating, requiring further investigation in large prospective studies. We also considered a range of covariates, including CVD risk factors, medications and physical activity. To reduce the risk of selection and misclassification bias, we included high‐quality objective administrative data from two sources including an 18‐year retrospective period (e.g. prevalent ASVD from ICD codes) and 14.5 years of prospective follow‐up. The ICD coding for dementia categorization also reduces potential bias in dementia diagnosis. Muscle function was also assessed using standardized assessments for hand grip strength and TUG. Nevertheless, limitations of this study must be acknowledged.

The observational nature of the study does not allow causality to be established. Furthermore, our results may not be generalized to other populations including younger women or men; thus, replication in other cohorts is essential. Although linked hospital discharge administrative data are reported to have high accuracy (96.7%), it has low sensitivity (21.2%) compared with chart review, therefore increasing dementia ascertainment compared with death certificates alone.^43^ Noteworthily, ICD‐10 dementia diagnoses from hospital records has substantial agreement (k = 0.71) with chart review including a sensitivity of 67% and a positive predictive value of 76%.^[S16]^ The incidence of dementia in the current study (18.5%) is similar to other studies in Australian women aged over 70 years followed over 16 years (20.4%)^44^ using multiple administrative data (e.g. aged care assessments data, hospital admissions, pharmaceutical data, death records and self‐reported survey data). Of importance, our results would have been biased towards the null if dementia diagnosis was overlooked. Given that women are at greater risk of developing late‐life dementia,^13^ it is important to elucidate the sex‐specific contribution of dementia risk factors, which could encompass a decline in physical function. However, as men and women have a different pattern of risk factors associated with grip strength decline,^45^ our findings may be less applicable to men. Additionally, as we adopted a Cox proportional hazards model, our hazard estimates are based on the first late‐life dementia event; it does not account for any further risk in women who may have presented with multiple dementia‐related hospitalizations. Further, severity was unable to be assessed, although dementia‐related hospitalizations are likely to be of a very serious nature as it warranted hospitalization. Low appendicular lean mass in conjunction with compromised strength/function are components of sarcopenia, a potential risk factor of interest. However, DXA‐derived lean mass was only obtained at year 1 in a small subset (~22.6%) of women. Due to compromised statistical power, we were unable to explore this further. Finally, we had limited power to assess the relationship between muscle function and dementia sub‐types. Vascular mechanisms are implicated in this association,^9^ but we did not have sufficient vascular dementia events to include in our analyses. Larger studies are needed to examine this relationship in women given that slow TUG was associated with unspecified late‐life dementia, but not AD events. However, the smaller AD sample may have contributed to this null finding.

In conclusion, our data indicate that muscle weakness and slower physical function, including their decline over 5 years, should be considered a risk factor for late‐life dementia in community‐dwelling older women. Despite current data indicating that functional assessment is not common in clinical practice,^46^ we provide further evidence for the importance of hand grip strength and TUG as potential clinical assessments to support early identification of community‐dwelling older women at risk of late‐life dementia. These findings are especially relevant to clinicians to direct high‐risk individuals during the asymptomatic prodromal period towards dementia‐related primary prevention programmes focusing on modifiable lifestyle factors (e.g. diet, exercise, smoking) to promote healthy ageing.

## Conflict of interest

All authors declare no conflicts of interest.

## Ethical approval

All participants provided written informed consent. Ethics approval was granted by the Human Ethics Committee of the University of Western Australia. CAIFOS and PLSAW studies were retrospectively registered on the Australian New Zealand Clinical Trials Registry (CAIFOS trial registration number #ACTRN12615000750583 and PLSAW trial registration number #ACTRN12617000640303) and complied with the Declaration of Helsinki. Human ethics approval for the use of linked data was provided by the Human Research Ethics Committee of the Western Australian Department of Health (project number #2009/24).

## Supporting information


**Table S1.** Baseline characteristics of included and excluded participants.
**Table S2.** Hazard ratios (95% CI) for any late‐life dementia events, hospitalizations and deaths over 14.5 years by weak hand grip strength and slow timed‐up‐and‐go.
**Table S3.** Multivariable‐adjusted hazard ratios (95% CI) including the individual variables that were used to compute the General Framingham Risk Score (age, body mass index, prevalent diabetes, systolic blood pressure) for late‐life dementia outcomes over 14.5 years by quartiles of hand grip strength and timed‐up‐and‐go (TUG).
**Table S4.** Competing risk (non‐dementia mortality) for late‐life dementia events by quartiles of hand grip strength and timed‐up‐and‐go (TUG).
**Table S5.** Multivariable‐adjusted hazard ratios (95% CI) for late‐life dementia events over 14.5 years by quartiles of hand grip strength and timed‐up‐and‐go (TUG) examined in women with or without the *Apolipoprotein E* ℇ4 (*APOE*_ℇ_4) genotype.
**Table S6.** Multivariable‐adjusted hazard ratios (95% CI) for any late‐life dementia event, hospitalizations and deaths over 9.5 years for the change in grip strength or timed‐up‐and‐go over 5 years (1998–2003) in 1052 women.
