# Supplementary material for: Impaired muscle function, including its decline, is related to greater long‐term late‐life dementia risk in older women
Source: J Cachexia Sarcopenia Muscle. 2023 Apr 19;14(3):1508–19. doi: 10.1002/jcsm.13227 (PMC10235875; doi:10.1002/jcsm.13227)
Supplement: Supplementary file 1 — Table S1. Baseline characteristics of included and excluded participants. Table S2. Hazard ratios (95% CI) for any late‐life dementia events, hospitalizations and deaths over 14.5 years by weak hand grip strength and slow timed‐up‐and‐go. Table S3. Multivariable‐adjusted hazard ratios (95% CI) including the individual variables that were used to compute the General Framingham Risk Score (age, body mass index, prevalent diabetes, systolic blood pressure) for late‐life dementia outcomes over 14.5 years by quartiles of hand grip strength and timed‐up‐and‐go (TUG). Table S4. Competing risk (non‐dementia mortality) for late‐life dementia events by quartiles of hand grip strength and timed‐up‐and‐go (TUG). Table S5. Multivariable‐adjusted hazard ratios (95% CI) for late‐life dementia events over 14.5 years by quartiles of hand grip strength and timed‐up‐and‐go (TUG) examined in women with or without the Apolipoprotein E ℇ4 (APOE ℇ4) genotype. Table S6. Multivariable‐adjusted hazard ratios (95% CI) for any late‐life dementia event, hospitalizations and deaths over 9.5 years for the change in grip strength or timed‐up‐and‐go over 5 years (1998–2003) in 1052 women. [file JCSM-14-1508-s001.docx]

**Supplementary References**

S1 Britt H, Scahill S, Miller G. ICPC PLUS for community health? A feasibility study. *Health Inf Manag*. 1997;27:171-5.

S2 Lewis JR, Lim W, Dhaliwal SS, Zhu K, Lim EM, Thompson PL, et al. Estimated glomerular filtration rate as an independent predictor of atherosclerotic vascular disease in older women. BMC Nephrology. 2012;13:58.

S3 Bruce DG, Devine A, Prince RL. Recreational physical activity levels in healthy older women: the importance of fear of falling. *J Am Geriatric Soc*. 2002;50:84-9.

S4 Hixson JE, Vernier DT. Restriction isotyping of human apolipoprotein E by gene amplification and cleavage with HhaI*. J Lip Res*. 1990;31:545-8.

S5 Wenham P, Price W, Blundell G. Apolipoprotein E genotyping by one-stage PCR. *The Lancet*. 1991;337:1158-9.

S6 D'Agostino RB, Sr., Vasan RS, Pencina MJ, Wolf PA, Cobain M, Massaro JM, et al. General cardiovascular risk profile for use in primary care: the Framingham Heart Study. *Circulation*. 2008;117:743-53.

S7 Zilkens RR, Spilsbury K, Bruce DG, Semmens JB. Clinical epidemiology and in-patient hospital use in the last year of life (1990–2005) of 29,884 Western Australians with dementia. *J Alzheimer's Disease*. 2009;17:399-407.

S8 Zilkens RR, Bruce DG, Duke J, Spilsbury K, Semmens JB. Severe psychiatric disorders in mid-life and risk of dementia in late- life (age 65-84 years): a population-based case-control study. *Curr Alzheimer Res*. 2014;11:681-93.

S9 Zilkens RR, Spilsbury K, Bruce DG, Semmens JB. Linkage of hospital and death records increased identification of dementia cases and death rate estimates. *Neuroepidemiology*. 2009;32(1):61-9.

S10 R Core Team. R: a language and environment for statistical computing. R Foundation for Statistical Computing, Vienna, Austra. URL http://www.R-project.org/. 2019.

S11 Fox J. Effect displays in R for generalised linear models. *J Stat Soft*. 2003;8:1-27.

S12 Harrell Jr F. rms: Regression Modeling Strategies. R package version 5.1–3. 1. 2019.

S13 Duchowny KA, Peterson MD, Clarke PJ. Cut points for clinical muscle weakness among older Americans. *Am J Prev Med*. 2017; 53(1):63-9.

S14 Zhu K, Devine A, Lewis JR, Dhaliwal SS, Prince RL. Timed up and go test and bone mineral density measurement for fracture prediction. *Arch Intern Med*. 2011;171(18):1655-61.

S15 Fine JPG, R.J. A proportional hazards model for the subdistribution of a competing risk. *J Am Stat Assoc*. 1999;94:496–509.

S16 Henderson T, Shepheard J, Sundararajan V. Quality of diagnosis and procedure coding in ICD-10 administrative data. *Med Care*. 2006;1011-9.

**Supplementary Table 1.** Baseline characteristics of included and excluded participants.

|  | **Included in the study** | **Excluded from the study** |
| --- | --- | --- |
| Number | 1225 | 235 |
| Age, years | 75.1 ± 2.7 | 75.3 ± 2.8 |
| Body mass index, kg/m^2a^ | 27.2 ± 4.6 | 27.3 ± 5.4 |
| Ever smoked, yes (%)^a^ | 440 (35.9) | 99 (42.1) |
| Systolic blood pressure, mmHg^b^ | 138 ± 18 | 139 ± 19 |
| Antihypertensive medication, yes (%) | 523 (42.7) | 113 (48.1) |
| Diabetes, yes (%) | 73 (6.0) | 22 (9.4) |
| Estimated CVD risk (Framingham) ^c^ | 22.1 ± 11.0 | 23.8 ± 12.1 |
| Statins medication, yes (%) | 234 (19.1) | 42 (17.9) |
| Low dose aspirin, yes (%) | 245 (22.0) | 63 (26.8) |
| Previous ASVD, yes (%) | 140 (11.4) | 38 (1.2) |
| Physical activity, Kcal/d^a^ | 113 (36-204) | 100 (0-200) |
| Alcohol, n (%)^d^ |  |  |
| None | 233 (19.0) | 64 (27.2) |
| < 10 standard drinks p/w | 792 (64.7) | 122 (51.9) |
| ≥ 10 standard drinks p/w | 200 (16.3) | 34 (14.5) |
| Randomisation, calcium (%) | 615 (50.2) | 115 (48.9) |
| *APOE* genotypes, yes (%)^e^ |  |  |
| *APOE* _ℇ_2/3 | 193 (15.8) | 11 (4.7) |
| *APOE* _ℇ_2/4 | 27 (2.2) | 2 (0.9) |
| *APOE* _ℇ_3/3 | 752 (61.4) | 41 (17.4) |
| *APOE* _ℇ_3/4 | 235 (19.2) | 16 (6.8) |
| *APOE* _ℇ_4/4 | 18 (1.5) | 6 (2.6) |
| Grip strength (kg)^f^ | 20.6 ± 4.6 | 19.9 ± 5.1 |
| Timed-up-and-go (s)^f^ | 9.3 (8.1-11.0) | 10.4 (8.4-12.2) |

Data expressed as mean ± SD or number and (%) for women who were included vs. excluded from the study due to missing data. Abbreviations: mmHg, millimetres mercury; ASVD, atherosclerotic vascular disease; *APOE*, Apolipoprotein E. For the following alphabets in superscript, sample sizes pertain only to women excluded from the study; ^a^ n=233, ^b^ n=185, ^c^ n=187, ^d^ n=220, ^e^ n=76, ^f^ n=226.

**Supplementary Table 2.** Hazard ratios (95% CI) for any late-life dementia events, hospitalizations and deaths over 14.5 years by weak hand grip strength and slow timed-up-and-go (TUG).

|  |  |  | **Hazard ratio (95%CI) for late-life dementia** | | |
| --- | --- | --- | --- | --- | --- |
|  |  |  | **Events** | **Hospitalizations** | **Deaths** |
| **Weak grip strength**  **(<22 kg)** | events, *n* (%) | 744 (60.7) | - | - | - |
|  | Unadjusted | - | **1.79 (1.32-2.42)** | **1.84 (1.33-2.54)** | **1.72 (1.07-2.77)** |
|  | Adjusted | - | **1.71 (1.26-2.32)** | **1.76 (1.27-2.49)** | **1.62 (1.00-2.62)** |
| **Slow TUG**  **(>10.2 sec)** | events, *n* (%) | 427 (34.8) | - | - | - |
|  | Unadjusted | - | **1.59 (1.21-2.10)** | **1.57 (1.17-2.11)** | **1.98 (1.28-3.05)** |
|  | Adjusted | - | **1.54 (1.16-2.04)** | **1.54 (1.14-2.07)** | **1.91 (1.23-2.97)** |
| **Poor muscle function^1^** | events, *n* (%) | 317 (25.9) | - | - | - |
|  | Unadjusted | - | **1.79 (1.34-2.38)** | **1.78 (1.31-2.41)** | **2.12 (1.36-3.31)** |
|  | Adjusted | - | **1.72 (1.28-2.31)** | **1.71 (1.27-2.38)** | **2.03 (1.29-3.21)** |

The referent group for each analysis are individuals who do not present with weak grip strength or slow TUG or a combination where appropriate. Multivariable-adjusted model includes general Framingham Risk Score, treatment code (calcium or placebo), alcohol intake, prevalent atherosclerotic vascular disease, prescription of statin medications, use of low dose aspirin, physical activity and Apolipoprotein E genotype. ^1^Poor muscle function categorised by presentation of both weak grip strength and slow TUG. Bolded indicates p<0.05 compared to quartile 4 for grip strength and quartile 1 for TUG.

**Supplementary Table 3.** Multivariable-adjusted hazard ratios (95% CI) including the individual variables that were used to compute the General Framingham Risk Score (age, body mass index, prevalent diabetes, systolic blood pressure) for late-life dementia outcomes over 14.5 years by quartiles of hand grip strength and timed-up-and-go (TUG).

|  |  |  | **Quartiles for grip strength or timed-up-and-go^1^** | | | |
| --- | --- | --- | --- | --- | --- | --- |
|  | Events |  | **Quartile 1** | **Quartile 2** | **Quartile 3** | **Quartile 4** |
| *Late-life dementia events* | *207 (16.9)* | *Grip strength* | **1.95 (1.32-2.89)** | **1.40 (1.02-1.93)** | 1.13 (0.88-1.45) | 1.0 (ref) |
|  |  | *TUG* | 1.0 (ref) | **1.44 (1.07-1.94)** | **1.83 (1.32-2.54)** | **2.17 (1.45-3.23)** |
|  |  |  |  |  |  |  |
| *Late-life dementia hospitalizations* | *183 (14.9)* | *Grip strength* | **1.98 (1.31-2.99)** | **1.39 (1.00-1.94)** | 1.09 (0.84-1.41) | 1.00 (ref) |
|  |  | *TUG* | 1.0 (ref) | **1.46 (1.06-2.00)** | **1.91 (1.35-2.71)** | **2.30 (1.50-3.51)** |
|  |  |  |  |  |  |  |
| *Late-life dementia deaths* | *83 (6.8)* | *Grip strength* | **2.04 (1.08-3.84)** | 1.45 (0.85-2.48) | 1.20 (0.77-1.89) | 1.00 (ref) |
|  |  | *TUG* | 1.0 (ref) | **1.91 (1.06-3.44)** | **2.34 (1.24-4.41)** | **2.63 (1.31-2.59)** |

^1^Estimated hazard ratios and 95% CI from Cox proportional hazards analysis comparing the median hand grip strength or TUG from each quartile compared to quartile 4 or 1, respectively. Median hand grip strength for quartile 1,2,3 and 4 was 16 kg, 19.5 kg, 22 kg and 25.8 kg, respectively. Median TUG for quartile 1,2,3 and 4 were 7.4 s, 8.7 s, 10.1 s and 12.4 s, respectively. Multivariable-adjusted model includes general age, body mass index, prevalent diabetes, systolic blood pressure, treatment code (calcium or placebo), alcohol intake, prevalent atherosclerotic vascular disease, prescription of statin medications, use of low dose aspirin, physical activity and Apolipoprotein E genotype. Bolded indicates p<0.05 compared to quartile 4 for grip strength and quartile 1 for TUG.

| **Supplementary Table 4.** Competing risk (non-dementia mortality) for late-life dementia events by quartiles of hand grip strength and timed-up-and-go (TUG). | | | |
| --- | --- | --- | --- |
|  | | **Unadjusted**  **SHR (95% CI)** | **Multivariable-adjusted**  **SHR (95% CI)^1^** |
| **Any late-life dementia event** | |  |  |
| Hand grip strength | Quartile 1, <17.5 kg | **2.72 (1.84-4.01)** | **2.63 (1.77-3.92)** |
|  | Quartile 2, 17.5 kg to <20.8 kg | 1.44 (0.94-2.20) | 1.41 (0.92-2.15) |
|  | Quartile 3, 20.8 kg to <23.2 kg | 1.36 (0.89-2.09) | 1.33 (0.87-2.03) |
|  | Quartile 4, ≥23.2 kg | 1.00 (ref) | 1.00 (ref) |
|  |  |  |  |
| Timed-up-and-go | Quartile 1, <8.1 s | 1.00 (ref) | 1.00 (ref) |
|  | Quartile 2, 8.1 s to <9.34 s | **1.70 (1.10-2.63)** | **1.68 (1.08-2.61)** |
|  | Quartile 3, 9.34 s to <10.99 s | **1.93 (1.26-2.97)** | **1.95 (1.26-3.01)** |
|  | Quartile 4, ≥10.99 s | **2.10 (1.37-3.21)** | **2.04 (1.32-3.14)** |
| SHR; sub-distribution hazard ratio. ^1^ Fine and Gray’s proportional sub-hazards analyses were adjusted General Framingham Risk Score plus treatment code (calcium or placebo), alcohol intake, prevalent atherosclerotic vascular disease, prescription of statin medications, use of low dose aspirin, physical activity and Apolipoprotein E genotype. Bolded indicate p<0.05 compared to quartile 4 or 1 for grip strength or TUG, respectively. | | | |

**Supplementary Table 5.** Multivariable-adjusted hazard ratios (95% CI) for late-life dementia events over 14.5 years by quartiles of hand grip strength and timed-up-and-go (TUG) examined in women with or without the *Apolipoprotein E* ℇ4 (*APOE* _ℇ_4) genotype.

|  |  | |  | | **Quartiles for grip strength or timed-up-and-go^1^** | | | | | | | |  |
| --- | --- | --- | --- | --- | --- | --- | --- | --- | --- | --- | --- | --- | --- |
|  | | Late-life dementia events | |  | | **Quartile 1** | | **Quartile 2** | | **Quartile 3** | | **Quartile 4** | |
| *APOE* _ℇ_4 *present*  *n=280* | | 68 (24.2) | | *Grip strength* | | **2.20 (1.14-4.25)** | | 1.32 (0.84-2.08) | | 1.02 (0.70-1.46) | | 1.00 (ref) | |
|  |  |  |  | *TUG* | | 1.0 (ref) | | **1.69 (1.00-2.83)** | | **1.97 (1.12-3.47)** | | **1.99 (1.01-3.93)** | |
|  | |  | |  | |  | |  | |  | |  | |
| *No APOE* _ℇ_4  *n=945* | | 139 (14.7) | | *Grip strength* | | **2.47 (1.53-3.99)** | | **1.82 (1.22-2.73)** | | **1.39 (1.01-1.92)** | | 1.00 (ref) | |
|  |  |  |  | *TUG* | | 1.0 (ref) | | 1.44 (0.99-2.08) | | **1.81 (1.21-2.71)** | | **2.12 (1.31-3.41)** | |
|  |  | |  | |  | |  | |  | |  | |  |

^1^Estimated hazard and 95% CI from Cox proportional hazards analysis comparing the median hand grip strength or TUG from each quartile compared to quartile 1 or 4, respectively. For women with APOE ℇ4, median hand grip strength and TUG for quartiles 1,2,3 and 4 were 15.5 kg, 20.0 kg, 22.0 kg, 25.5 kg, and 7.4 s, 8.7 s, 10.0 s and 12.2 s, respectively. For women without *APOE* _ℇ_4, median hand grip strength and TUG for quartiles 1,2,3 and 4 were 16 kg, 19.5 kg, 22 kg, 25.8 kg, and 7.4 s, 8.7 s, 10.1 s and 12.4 s, respectively. Multivariable-adjusted model includes Framingham Risk Score plus treatment code (calcium or placebo), alcohol intake, prevalent atherosclerotic vascular disease, prescription of statin medications, use of low dose aspirin and physical activity. Bolded indicates p<0.05 compared to quartile 4 or 1 for grip strength or TUG, respectively, where appropriate.

**Supplementary Table 6.** Multivariable-adjusted hazard ratios (95% CI) for any late-life dementia event, hospitalizations and deaths over 9.5 years for the change in grip strength or timed-up-and-go (TUG) over 5 years (1998-2003) in 1052 women.

|  |  | **Hazard ratio (95% CI) for late-life dementia** | | |
| --- | --- | --- | --- | --- |
|  |  | **Events** | **Hospitalizations** | **Deaths** |
| *Grip strength*  *Per kg decrease*  *over 5 years* | *Adjusted* | **1.07 (1.03-1.11)** | **1.08 (1.04-1.13)** | **1.07 (1.00-1.14)** |
|  |  |  |  |  |
| *Timed-up-and-go*  *Per sec increase*  *over 5 years* | *Adjusted* | **1.07 (1.03-1.11)** | **1.07 (1.02-1.11)** | **1.10 (1.03-1.16)** |

Multivariable-adjusted model includes general Framingham Risk Score, treatment code (calcium or placebo), alcohol intake, prevalent atherosclerotic vascular disease, prescription of statin medications, use of low dose aspirin, physical activity and Apolipoprotein E genotype and baseline grip strength or TUG (where appropriate).
